# Anti-Nociceptive Effects of Sphingomyelinase and Methyl-Beta-Cyclodextrin in the Icilin-Induced Mouse Pain Model

**DOI:** 10.3390/ijms25094637

**Published:** 2024-04-24

**Authors:** Ádám Horváth, Anita Steib, Andrea Nehr-Majoros, Boglárka Kántás, Ágnes Király, Márk Racskó, Balázs István Tóth, Eszter Szánti-Pintér, Eva Kudová, Rita Skoda-Földes, Zsuzsanna Helyes, Éva Szőke

**Affiliations:** 1 Department of Pharmacology and Pharmacotherapy, Medical School, University of Pécs, Szigeti Str. 12., H-7624 Pécs, Hungary; horvath.adam2@pte.hu (Á.H.); steib.anita@pte.hu (A.S.); majoros.andii@gmail.com (A.N.-M.); boglarka.kantas@aok.pte.hu (B.K.); kiraly.agnes19@gmail.com (Á.K.); helyes.zsuzsanna@pte.hu (Z.H.); 2 Department of Pharmacology, Faculty of Pharmacy, University of Pécs, Rókus Str. 2., H-7624 Pécs, Hungary; 3 National Laboratory for Drug Research and Development, Magyar Tudósok Cct. 2., H-1117 Budapest, Hungary; 4 Hungarian Research Network, Chronic Pain Research Group, University of Pécs, Szigeti Str. 12., H-7624 Pécs, Hungary; 5 Department of Obstetrics and Gynaecology, University of Pécs, Édesanyák Str. 17., H-7624 Pécs, Hungary; 6 Department of Physiology, Faculty of Medicine, University of Debrecen, Nagyerdei Cct. 98., H-4032 Debrecen, Hungary; racsko.mark@med.unideb.hu (M.R.); toth.istvan@med.unideb.hu (B.I.T.); 7 Institute of Organic Chemistry and Biochemistry of the Czech Academy of Sciences, Flemingovo Namesti 2, 166 10 Prague, Czech Republic; eszter.szanti-pinter@uochb.cas.cz (E.S.-P.); kudova@uochb.cas.cz (E.K.); 8 Institute of Chemistry, Department of Organic Chemistry, University of Pannonia, Egyetem Str. 10., H-8200 Veszprém, Hungary; skodane.foldes.rita@mk.uni-pannon.hu; 9 PharmInVivo Ltd., Szondy György Str. 10., H-7629 Pécs, Hungary

**Keywords:** cholesterol, lipid raft, methyl-beta-cyclodextrin, pain, sphingomyelinase, Transient Receptor Potential

## Abstract

The thermo- and pain-sensitive Transient Receptor Potential Melastatin 3 and 8 (TRPM3 and TRPM8) ion channels are functionally associated in the lipid rafts of the plasma membrane. We have already described that cholesterol and sphingomyelin depletion, or inhibition of sphingolipid biosynthesis decreased the TRPM8 but not the TRPM3 channel opening on cultured sensory neurons. We aimed to test the effects of lipid raft disruptors on channel activation on TRPM3- and TRPM8-expressing HEK293T cells in vitro, as well as their potential analgesic actions in TRPM3 and TRPM8 channel activation involving acute pain models in mice. CHO cell viability was examined after lipid raft disruptor treatments and their effects on channel activation on channel expressing HEK293T cells by measurement of cytoplasmic Ca^2+^ concentration were monitored. The effects of treatments were investigated in Pregnenolone-Sulphate-CIM-0216-evoked and icilin-induced acute nocifensive pain models in mice. Cholesterol depletion decreased CHO cell viability. Sphingomyelinase and methyl-beta-cyclodextrin reduced the duration of icilin-evoked nocifensive behavior, while lipid raft disruptors did not inhibit the activity of recombinant TRPM3 and TRPM8. We conclude that depletion of sphingomyelin or cholesterol from rafts can modulate the function of native TRPM8 receptors. Furthermore, sphingolipid cleavage provided superiority over cholesterol depletion, and this method can open novel possibilities in the management of different pain conditions.

## 1. Introduction

Studies about lipid rafts represent a hot topic in the biological and pharmacological field for the past two to three decades. The original conception of lipid rafts was first introduced by Karnovsky and co-workers [1]. Lipid rafts are specialized microdomains of the plasma membrane, which are rich in cholesterol, sphingolipids, and gangliosides [2]. These platforms have not only special physicochemical features but also play pivotal roles in several biological and pathological processes, such as signal transduction, tumor formation, and immunological responses [3,4,5,6,7]. It is also well-known that several receptors and ion channels are functionally associated with the raft regions. Among others, the gamma-aminobutyric acid (GABA receptor), 2-amino-3-(5-methyl-3-oxo-1,2-oxazol-4-yl)-propanoic-acid (AMPA) glutamate receptor, nicotinic acetylcholine receptor, and several members of the Transient Receptor Potential (TRP) ion channel family are located in lipid rafts [8,9,10,11].

The integrity of the rafts can be interrupted in several ways. The two main components of the lipid rafts are cholesterol and sphingolipids, which can be depleted from the raft regions. For cholesterol depletion, the gold standard is methyl-beta-cyclodextrin (MCD), which is a complex-forming agent [12]. For cholesterol depletion, our research group synthesized a novel carboxamido-steroid compound (C1) [13,14]. The sphingolipid content can be decreased by the inhibition of de novo synthesis with myriocin (Myr) [15]. The hydrolysis of sphingomyelins (SM) to phosphocholine and ceramide by sphingomyelinase (SMase) is another possibility of sphingomyelin depletion [16].

Since several studies showed that TRP receptors are located in the lipid raft region, our research group aimed to investigate the functional connection between lipid rafts and some pain-related members of this ion channel family. TRP Melastatin (TRPM) channels, such as TRPM3 and TRPM8, are heat-sensitive receptors and play a pivotal role in pain modulation [17,18,19]. Both channels have structural similarities to other TRP receptors; however, their function and gating mechanisms are different [20]. TRPM3 can be activated by noxious heat, as well as by the neurosteroid pregnenolone sulphate (20-oxo-pregn-5-en-3β-yl sulphate, PS) and CIM-0216 [21,22,23,24]. TRPM8 can be activated by cold temperatures and cooling agents, such as menthol or the synthetic icilin compound [25,26,27]. Morenilla-Palao and co-workers showed that lipid raft segregation by 10 mM MCD shifted the TRPM8 activation threshold to higher temperatures [28]. We have already described that cholesterol depletion by MCD, depletion of SM by SMase, and the inhibition of ganglioside biosynthesis using Myr significantly and dose-dependently decreased the TRPM8 but not the TRPM3 cation channel opening on cultured trigeminal ganglion (TG) neurons. Only treatment with C1 (100 µM) could decrease the activation of TRPM3 ion channels [29].

Furthermore, in our previous studies, we demonstrated the inhibitory effects of lipid raft disruptor compounds on the activation of the most studied members of the TRP superfamily–TRP Vanilloid 1 (TRPV1) and TRP Ankyrin 1 (TRPA1). We described that sphingolipid depletion by SMase or Myr, and cholesterol depletion by MCD or C1 decreased the agonist-induced activation of the ion channels in vitro, and these inhibitory effects can lead to antinociception in in vivo models [29,30,31,32,33]. Besides us, a couple of other groups investigated the connection between lipid rafts and TRP channels, but most of these studies, such as the work of Liu and co-workers and Startek and co-workers, are focused on in vitro features [10,34,35]. Only a few in vivo experiments provided data about the in vivo effects of lipid raft disruption, and these studies focused on only cholesterol depletion [36,37,38]. 

Based on the previous results related to TRPV1 and TRPA1 channels and the in vitro described potential functional interaction of the lipid rafts and TRPM channels, we aimed to study the safety profile of lipid raft disruptors on native Chinese Hamster Ovary (CHO) cell lines using cell viability assays. We also aimed to test their effects on channel activation on TRPM3 and TRPM8 expressing Human Embryonic Kidney 293T (HEK293T) cells in vitro, as well as their potential antinociceptive effects in TRPM3 and TRPM8-related acute pain models in mice. 

## 2. Results

### 2.1. Cholesterol Depletion Decreases Cell Viability in a Concentration-Dependent Manner

**Figure 1:**
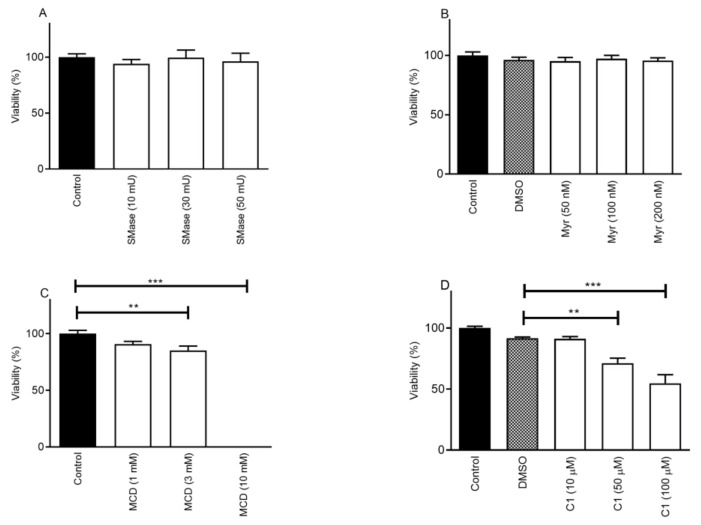
Effect of lipid raft disruptors on cell viability. The effect of SMase (**A**), Myr (**B**), MCD (**C**), and C1 (**D**) on viability is presented. Data are presented as means ± standard error of mean (SEM), n = 9; three independent experiments in triplicates. Statistical analysis was performed using one-way Analysis of Variance (ANOVA) followed by Dunnett’s post hoc test. (** *p* < 0.01; *** *p* < 0.001 MCD/C1 samples vs. saline control/DMSO control).

First, we investigated the effects of sphingolipid-depleting agents. Neither SMase (10, 30, and 50 mU) nor Myr (50 nM, 100 nM, and 200 nM) treatment changed the viability of CHO cells compared to the control samples after 24 h. In the case of SMase, the corresponding values were the following: control–100.0 ± 3.0%; 10 mU–94.0 ± 3.9%; 30 mU–99.5 ± 6.8%, and 50 mU–96.3 ± 7.2% (Figure 1A). In the case of Myr, a dimethyl sulfoxide (DMSO) control (DMSO content was 0.004%) was used to compare the values with 50 nM, 100 nM, and 200 nM Myr. These values were 96.2 ± 2.3%; 95.2 ± 3.1%; 97.3 ± 2.8%, and 95.7 ± 2.4% in the DMSO and treated samples, respectively (Figure 1B). 

During the examination of cholesterol depletion, cells were treated with 1 mM, 3 mM, and 10 mM concentrations of MCD for 24 h. The percentage of viable cells showed a concentration-dependent decrease in the presence of 3 and 10 mM MCD. The corresponding values in the groups were the following: control–100.0 ± 3.0%; 1 mM–90.8 ± 2.5%; 3 mM–85.2 ± 3.9%, and 10 mM–0.1 ± 0.007% (Figure 1C). In the presence of 10 mM MCD, the cells were not viable. C1 treatment (10 µM, 50 µM, and 100 µM) for 24 h showed similar effects as the MCD treatment; however, it was less pronounced. The viability values were compared to a DMSO control (DMSO content was 1%). The viability was 91.7 ± 1.0%; 91.3 ± 1.7%; 71.3 ± 4.1%, and 54.7 ± 7.1% in the DMSO, 10 µM, 50 µM, and 100 µM C1 samples, respectively (Figure 1D).

### 2.2. SMase and MCD Reduced the Duration of Icilin-Induced Nocifensive Behavior

**Figure 2:**
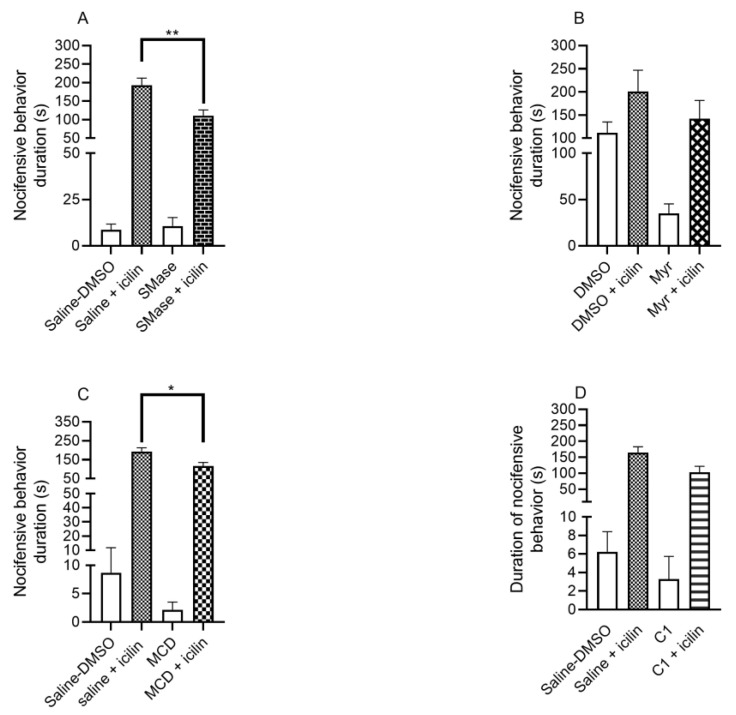
Effect of lipid raft disruptors on icilin-induced acute nocifensive pain. The effect of 50 mU SMase (**A**), 1 mM Myr (**B**), 15 mM MCD (**C**), and 100 µM C1 compound (**D**) on nocifensive behavior is presented. Data are presented as means ± SEM, n = 6–27 mice/group. Statistical analysis was performed using one-way ANOVA followed by Bonferroni’s post hoc test. (* *p* < 0.05; ** *p* < 0.01; SMase pretreated group vs. saline pretreated group).

In the icilin-induced acute nocifensive pain model, we investigated the effects of sphingolipid-depleting agents. SMase significantly reduced the duration of the nocifensive reaction. In the case of 50 mU SMase, during the 20 min observation period, icilin induced 193.0 ± 19.6 s long pain reaction in the control group, while in the SMase pretreated group, the corresponding value was 110.8 ± 15.6 s (Figure 2A). The treatment with 1 mM Myr compared to the DMSO-treated control did not significantly alter the duration of pain. One hundred percent DMSO induced a 200.9 ± 45.8 s long nocifensive reaction, while in the Myr-pretreated group, the measured value was 142.4 ± 39.3 s (Figure 2B).

The effect of cholesterol-depleting compounds was also investigated. MCD significantly diminished the duration of the nocifensive reaction. In the case of 15 mM MCD, the measured values were the following in the control and MCD pretreated groups, respectively: 193.0 ± 19.6 s vs. 115.6 ± 19.9 s (Figure 2C). C1 compound in 100 µM concentration did not induce a significant change in the duration of the pain reaction. The values were the following: 165.0 ± 17.8 s in the control group, while 102.8 ± 18.6 s in the C1 pretreated group (Figure 2D).

### 2.3. Effect of Carboxamido-Steroid Compound C1 on Acute Nocifensive Pain Reaction Induced by PS and CIM-0216

**Figure 3:**
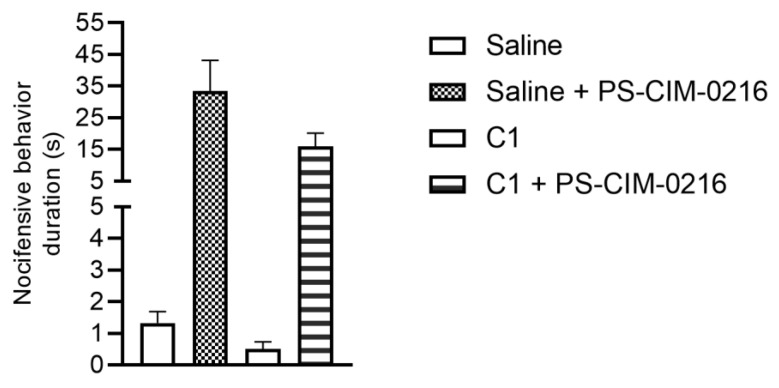
Effect of 100 µM C1 on PS and CIM-0216-induced acute nocifensive pain. Data are presented as means ± SEM, n = 5–8 mice/group. Statistical analysis was performed using a one-way ANOVA followed by Bonferroni’s post hoc test.

In this model, we investigated the effect of C1 based on our previous in vitro findings on TG neurons, whereby C1 inhibited the activation of TRPM3 channels using ratiometric Ca-imaging methods [29]. The combination of PS with CIM-0216 (both in 10 µM) can enhance the effect of PS [24]. In the saline pretreated group, we measured 33.5 ± 9.7 s long nocifensive reactions, while in the 100 µM C1 pretreated group, the duration of pain response was 16.0 ± 4.2 s (Figure 3). Administration of C1 did not change the duration significantly.

### 2.4. Lipid Raft Disruptors Do Not Inhibit the Activity of Recombinant TRPM3 and TRPM8

**Figure 4:**
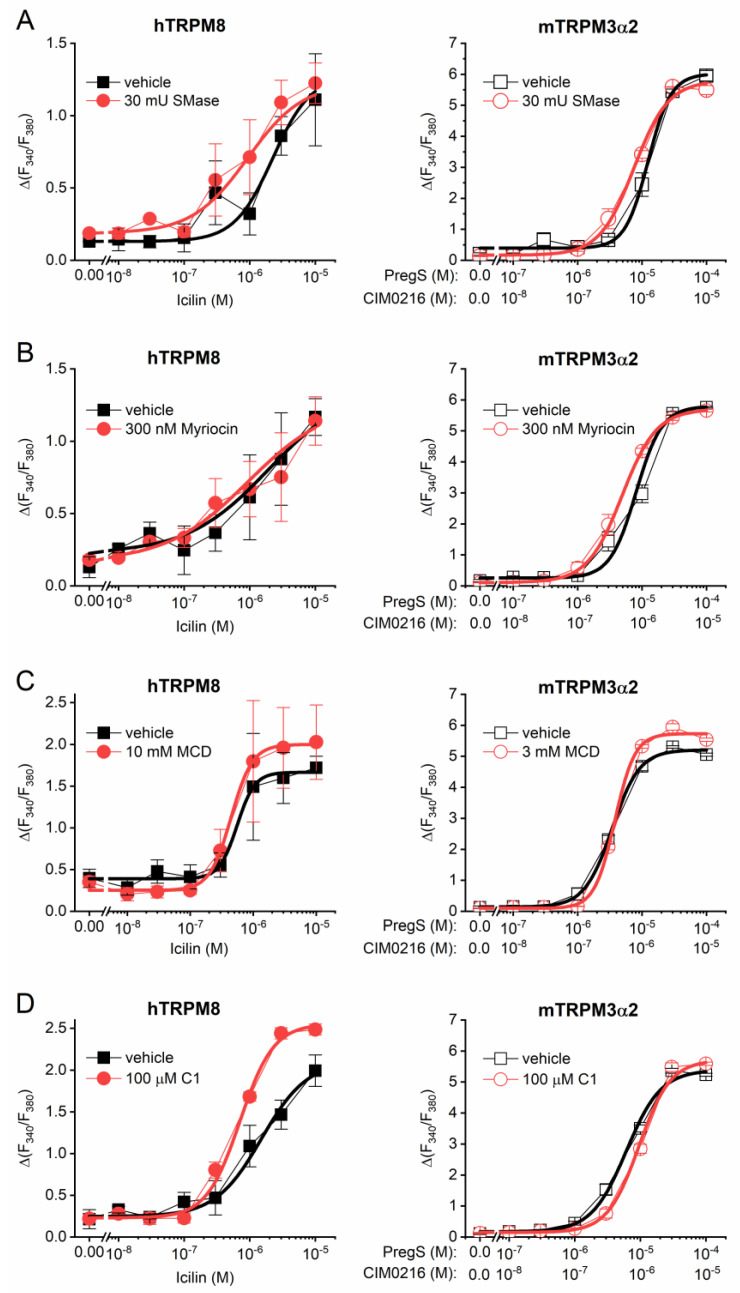
Effect of lipid raft disruptors on the agonist-induced activity of TRPM8 and TRPM3. HEK293T cells overexpressing either human TRPM8 (hTRPM8) or TRPM3α2 variant of mouse TRPM3 (mTRPM3α2) were pretreated with (**A**) SMase, (**B**) Myriocin, (**C**) MCD, and (**D**) C1 compound and subjected to [Ca^2+^]_IC_ measurements upon applying various concentrations of agonists, as indicated in the figure. Each data point is presented as the mean ± SEM of three independent measurements (n = 3). Concentration–response curves were fitted as described in the Section 4.

Finally, we investigated the effect of lipid raft disruptors on the activity of recombinant TRPM8 and TRPM3 expressed in HEK293T cells in intracellular Ca^2+^ ([Ca^2+^]_IC_) measurements. In line with the in vivo experiments, icilin and PS/CIM-0216 (10:1) were applied to activate TRPM8 and TRPM3, respectively. Neither acute application of SMase (30 mU for 45 min), nor 24 h-long pretreatment with Myr (300 nM) inhibited the dose-dependent activation of the recombinant TRPM8 and TRPM3 (Figure 4A,B and Table 1). Cholesterol-depleting MCD (3 or 10 mM for 45 min) or C1 (100 µM for 45 min) were also ineffective in decreasing the agonist-induced Ca^2+^ signals (Figure 4C,D and Table 1). Interestingly, acute application of 10 mM MCD was found to be cytotoxic on TRPM3-expressing cells, which was indicated by elevated basal [Ca^2+^]_IC_ and diminished responses for ionomycin applied as a positive control. Therefore, TRPM3-expressing cells were pretreated with 3 mM MCD instead of 10 mM applied on TRPM8-expressing cells. These results suggest that the regulation of the recombinant receptors by lipid raft components in HEK cells may differ from that of the native receptor.

## 3. Discussion

We demonstrate here the anti-nociceptive effect of lipid raft disruption via SM hydrolysis by SMase and cholesterol depletion by MCD on icilin-induced nocifensive behavior in mice. We also demonstrate that sphingolipid depletion by SMase and Myr does not reduce cell viability and, therefore, can be safe upon in vivo administration. In contrast, long-term (≥24 h) application of MCD and C1 decreases viability.

Previously, we described that TRPV1, TRPA1, and TRPM8 receptor activation was inhibited after lipid raft disruption in both TG neurons and TRPV1- or TRPA1-transfected CHO cells by cholesterol depletion with MCD and C1 compound, by SM cleavage with SMase or by sphingolipid biosynthesis inhibition with Myr [29,30,31]. Furthermore, we showed cholesterol depletion from the plasma membrane of CHO cells and TG neurons after MCD and the carboxamido-steroid compound C1 treatment by decreased staining with cholesterol indicator filipin and fluorescence spectroscopy [29,31]. In TG neurons, the lipid raft depletor compounds diminished the capsaicin (TRPV1), allyl isothiocyanate or formaldehyde (TRPA1), and icilin (TRPM8)-induced Ca^2+^-influx [30,31]. However, lipid raft disruption by MCD increased menthol-induced activation of TRPM8 on transfected cell lines and mouse dorsal root ganglion neurons [28]. Only our C1 compound was able to decrease the PS-evoked TRPM3 activation on TG neurons [29]. There are only a few in vivo animal experiments to prove that cholesterol depletion results in an analgesic effect. We have already described that MCD and C1 had antinociceptive effects via inhibiting the TRPV1 and TPRA1 ion channel activation in mice in distinct pain mechanisms [32]. The role of cholesterol content in the plasma membrane in TRP channel activation and the potential significance of cholesterol-TRP interactions were also proposed by other researchers [34,35]. Cryo-electron microscopy data supported the importance of the lipids on conformation changes between active, inactive, or desensitized states of TRP ion channels [39]. These data confirmed our hypothesis that direct protein–lipid hydrophobic interactions were important in channel activation and inhibition. TRP channel inhibition was due to a change in their position in the plasma membrane making the binding sites unavailable for agonists [40]. The role of ganglioside depletion in analgesia has been reviewed [41]. 

The significance of sphingolipids in pain sensation via the modulation of TRP channel activation is poorly understood and we had only in vitro experimental results [30,31,35]. Our research group provided the first in vivo data that intraplantar injection of SMase and Myr reduced TRPV1 and TRPA1-related pain intensity in several animal models [32,33]. The present study provides the first in vivo results on the analgesic effect of SMase and MCD on icilin-evoked pain by SM hydrolyzation or cholesterol depletion. The duration of nocifensive behavior after icilin injection into the hind paw was significantly decreased by both compounds. Although our previous in vitro data showed that Myr-evoked sphingolipid synthesis inhibition and C1 reduced icilin-evoked Ca^2+^-influx in TG neurons [31], they did not influence the duration of acute nocifensive reaction in mice. We have previously described that SMase treatment had several advantages over cholesterol depletion or Myr treatment. It has a localized action on the plasma membrane, its effect develops within 30 min, and the known metabolites of SMase can also be examined to exclude their potential interference with the observed reactions [31]. Moreover, in the present study, we demonstrated that SMase, like Myr, did not decrease cell viability determined using a frequently used assay, which is based on the ATP level in living cells.

The carboxamido-steroid compound C1, but not MCD, SMase, or Myr, was able to diminish PS-induced TRPM3 activation on native TG neurons in our previous experiments [29]. From a structural point of view, the TRPM3 ion channel is a unique TRP channel with specific features [21,42]. The neurosteroids PS, epipregnanolone sulphate, and dihydro-D-erythro-sphingosine can activate this ion channel via a specific steroid binding site on the channel [42,43,44]. Controversial results have also been described in previous studies, MCD increased the TRPM3 channel activation by PS on contractile and proliferating phenotypes of mouse vascular smooth muscle cells, while an MCD/cholesterol complex administration decreased human and mouse TRPM3 activation [42,45]. TRPM3 ion channel together with a presently unknown auxiliary protein might compose a quaternary complex, and the ion channel in this form is quite strong to oppose the MCD-caused lipid raft disruption as Drews and co-workers suggest [42]. Based on our previous in vitro observations, the inhibition of TRPM3 activation in an in vivo animal model was performed only with C1. Although, under in vitro circumstances, C1 was able to reduce the PS-evoked TRPM3 activation on TG neurons [29], it had no analgesic effect in the PS + CIM-0216-induced pain model. Moreover, in our experiments, neither cholesterol depletion by MCD and C1 treatment, nor sphingolipid depletion by SMase and Myr decreased the TRPM8 and TRPM3 activation in receptor-expressing HEK293T cells. We suggest that damage in the lipid environment is not sufficient to abolish the activation of overexpressed ion channels in HEK cells and the regulation of the recombinant receptors by lipid raft components may differ from that of the native receptor. Differences between receptor actions in the lipid raft compartments of HEK293T cells and sensory neurons have been described. In P2X3 purinoceptor knock-in sensory neurons the lipid rafts were more abundant and facilitated P2X3-mediated responses, but MCD treatment did not change the function of the recombinant P2X3 receptors expressed by HEK293T cells [46]. It is concluded that the results obtained in the mouse models are of great importance regarding the in vivo anti-nociceptive effects of lipid raft disruption. This might suggest the peripheral analgesic potential of SMase and MCD after local administration.

## 4. Materials and Methods

### 4.1. Animals and Ethics

Twelve to sixteen weeks-old male NMRI mice were used in the icilin and PS-induced acute pain models. Only male mice were used in the present study because the menstrual cycle of female animals can modulate pain responses [47]. The animals were kept in standard plastic cages at 24–25 °C, under a 12–12 h light-dark cycle, and provided with standard rodent chow and water ad libitum in the Laboratory Animal House of the Department of Pharmacology and Pharmacotherapy, University of Pécs. All experimental procedures were carried out according to the 1998/XXVIII Act of the Hungarian Parliament on Animal Protection and Consideration Decree of Scientific Procedures of Animal Experiments (243/1988). The studies were approved by the Ethics Committee on Animal Research of Pécs University according to the Ethical Codex of Animal Experiments and the license was given (license No.: BA02/2000-33/2022).

### 4.2. Cell Culture

Native CHO cells were maintained in complete Dulbecco’s Modified Eagle’s Medium (DMEM) (500 mL DMEM Low Glucose supplemented with 50 mL FBS, 10 mL GlutaMAXTM-I, 10 mL MEM Non-Essential Amino Acid Solution and 500 µL Pen/Strep Mixture) in T-25 flasks at 37 °C in a 5% CO_2_ incubator.

HEK293T cells stably overexpressing the hTRPM8 and mTRPM3α2 were provided as a kind gift by Prof. Thomas Voets (KU Leuven, Leuven, Belgium) and cultured as described before [48,49]. Briefly, cells were cultured in DMEM medium supplemented with 10% fetal bovine serum, 50 U/mL penicillin, 50 μg/mL streptomycin, 10 mM Glutamax, Non-Essential-Amino-Acids (all from ThermoFisher, Waltham, MA, USA), and 500 μg/mL G418 (Sigma-Aldrich, St. Louis, MO, USA) for HEK-hM8 or 200 μg/mL Hygromycin (ThermoFisher) for HEK-mM3α2.

### 4.3. Cell Viability Assay

To determine the number of viable CHO cells after compound treatment CellTiter-Glo^®^ Luminescent Cell Viability Assay measuring the amount of intracellular adenosine triphosphate (ATP) was performed according to manufacturer’s protocol. Briefly, cells in culture media were seeded to opaque-walled 96-well plates (5000 cells/well) and incubated overnight. The next day, DMEM was aspired from the cells, and cells were treated with increasing concentrations of MCD (1, 3, 10 mM), C1 (50, 100, 200 nM), Myr (50, 100, 200 nM), or SMase (10, 30, 50 mU) in 100 µL DMEM. Untreated cells served as control and DMEM as no-cell control. In the case of C1 and Myr, a DMSO control was measured. Following 24 h incubation, plates were equilibrated to room temperature and 100 µL CellTiter-Glo^®^ reagent was added to each well, which already contained 100 µL medium. Plates were placed on an orbital shaker for 2 min followed by 10 min incubation at room temperature. Luminescence was measured in a microplate reader (EnSpire AlphaLISA, PerkinElmer Inc.—Waltham, MA, USA) and luminescence values were normalized to untreated control. Cell viability percentage was calculated by comparing the luminescence values of treated wells to untreated control cells. All experiments were performed in three independent measurements with triplicates from each sample.

### 4.4. Icilin-Induced Acute Nocifensive Pain Reaction

The effect of lipid raft disruptors compared to vehicle controls was investigated in a TRPM8-related pain model induced using the TRPM8 agonist icilin. Intraplantar pretreatment (20 μL) was performed with 50 mU SMase, 1 mM Myr, 100 μM C1, and 15 mM MCD. Thirty minutes later, icilin was injected intraplantarly (20 μL, 2.4 mg/mL) in the right hind paw which immediately induced nocifensive reactions (e.g., hind paw licking, lifting, shaking, biting). The duration of these behavior reactions was measured constantly for 20 min [50].

### 4.5. PS-CIM-0216 Evoked Acute Nocifensive Pain Reaction

To investigate the effect of lipid raft disruptors in a TRPM3-mediated pain model, mice were intraplantarly pretreated with 50 mU SMase, 1 mM Myr, 100 μM C1, and 15 mM MCD. After 30 min, TRPM3 agonist PS and CIM-0216 (20 μL in equimolar concentration) were injected intraplantarly to induce nocifensive behavior like hind paw licking, lifting, shaking, and holding. These reactions were measured for 3 min [24]. 

### 4.6. Fluorescent Ca^2+^ Measurements

Fluorescent measurement of [Ca^2+^]_IC_ in HEK293T cells was performed using Fura-2 AM fluorescent Ca^2+^ indicator dye according to our previously optimized protocol [49]. Cells were seeded in 96-well black wall/clear bottom plates (Greiner Bio-One, Frickenhausen, Germany) previously coated with Poly-L-Lysine HBr (Sigma-Aldrich, St. Louis, MO, USA) at a density of 100,000 cells per well in normal cell culture medium. The next day, cells were loaded with 2 μM Fura-2-AM (ThermoFisher, Waltham, MA, USA) solved in culture medium at 37 °C for 45 min and washed three times with Ca^2+^-buffer (150 mM NaCl, 5 mM KCl, 1 mM MgCl_2_ × 6H_2_O, 2 mM CaCl_2_ × 2H_2_O, 10 mM glucose × H_2_O, 10 mM HEPES, pH 7.4 (all from Sigma-Aldrich)). Lipid raft disruptor agents were applied during Fura-2 loading except for Myriocin applied for 24 h. Measurements were carried out using a FlexStation 3 fluorescent microplate reader (Molecular Devices, Sunnyvale, CA, USA) and cytoplasmic Ca^2+^ concentration (reflected by the ratio of fluorescence measured at λEX1: 340 nm, λEX2: 380 nm, λEM: 510 nm (F340/F380)) was monitored during application of agonists in various concentrations. At the end of each measurement, 3 μM ionomycin was applied as positive control. During the measurements, cells in a given well were exposed to only one given concentration of the agents. Measurements were carried out at ambient temperature.

Data were plotted as mean ± SEM and logistic dose–response curves were fitted using the equation:

$$y=\frac{A2+\left(A1-A2\right)}{\left(1+{\left(\;\frac{X}{X0}\right)}^{p}\right)}$$

where the calculated parameters are: A1: initial value (ymin), A2: final value (ymax), x0: center (EC50) and *p* is the calculated power. 

### 4.7. Drugs and Chemicals

DMEM Low Glucose (1 g/L), with L-Glutamine, with Sodium Pyruvate (Capricorn Scientific GmbH—Ebsdorfergrund, Germany) Fetal Bovine Serum (FBS) (EuroClone SpA—Pero, Italy) GibcoTM GlutaMAXTM-I (100×) (Thermo Fisher Scientific Inc.) GibcoTM MEM Non-Essential Amino Acid Solution (100×) (Thermo Fisher Scientific Inc.) Penicillin-Streptomycin Mixture (Pen/Strep) (Lonza—Basel, Switzerland) Trypsin/EDTA Solution (10×) (Lonza) Phosphate Buffer Saline (PBS): NaCl, KCl, Na_2_HPO_4_, KH_2_PO_4_ (Molar Chemicals Ltd.—Halásztelek, Hungary) CellTiter-Glo^®^ Luminescent Cell Viability Assay (Promega Corporation—Madison, WI, USA). All the other compounds–except CIM-0216 (Tocris Bioscience—Bristol, UK) were purchased from Sigma, St. Louis, MO USA. MCD was dissolved in saline to reach a final concentration of 15 mM. C1 was dissolved in DMSO to obtain a 10 mM stock solution. Further dilution was made with saline to reach final concentrations of 100 μM. Myr from *Mycelia sterilia* was dissolved in DMSO to obtain a 5 mM stock solution. Further dilution was made with DMSO to reach the final concentration of 1 mM (DMSO content was 100% in the final solution). SMase from *Bacillus cereus* was purchased in a glycerol-buffered solution, and further dilution was made with saline to reach the concentrations of 50 mU. Icilin was dissolved in DMSO to obtain 10 mg/mL stock and it was further diluted to 2.4 mg/mL concentration with saline (DMSO content was 24% in the final solution). PS and CIM-0216 were dissolved in DMSO to obtain 10 mM concentration, and further diluted with saline to reach a final concentration of 1 mM (DMSO content was 10% in the final solution). 

### 4.8. Statistical Analysis

The in vitro data were statistically analyzed using GraphPad Prism 8. After confirming normal distribution with a D’Agostino-Pearson test, a one-way ANOVA was performed followed by Dunnett’s post hoc test. Results are presented as mean ± SEM. All in vivo data are means ± SEM of at least 5 animals per group. Statistical analysis was performed using a one-way ANOVA followed by Bonferroni’s multiple comparisons post hoc test, in all cases *p* < 0.05 was considered statistically significant. Data from the fluorescent Ca^2+^ measurements were analyzed and fitted using Origin 2018 (OriginLab Corporation, Northampton, MA, USA).

## 5. Conclusions

Based on our findings, we conclude that the lipid membrane environment by sphingolipid or cholesterol depletion modulates the function of TRPM8 ion channels, which are involved in thermosensation and pain. Our in vivo results suggest that membrane sphingolipid modification, particularly by SMase, which has no toxic effect on the cellular environment, might open novel analgesic opportunities. 

## Figures and Tables

**Table 1:** Effect of lipid raft disruptors on the agonist-induced activity of recombinant TRPM8 and TRPM3. Efficacy (EC_50_) and effectivity (maximal response) of the agonists were determined based on dose–response curves recorded in [Ca^2+^]_IC_ measurements.

Pretreatment	hTRPM8		mTRPM3α2	
	Δ(pEC50) of Icilin	Maximal Response to Icilin (Vehicle Pretreated Control = 1)	Δ(pEC50) of PS *	Maximal Response to PS/CIM-0216 (10:1) (Vehicle Pretreated Control = 1)
30 mU SMase	0.38	0.96	0.22	0.96
300 nM Myriocin	0.31	0.92	0.22	0.98
3 mM MCD	-	-	−0.04	1.10
10 mM MCD	0.24	1.14	-	-
100 µM C1	0.33	1.21	−0.19	1.06

* in the presence of CIM-0216 (PS:CIM-0216 = 10:1).

## Data Availability

The original data presented in the study are included in the article. Further inquiries can be directed to the corresponding author.

## Abbreviations

AMPA	2-amino-3-(5-methyl-3-oxo-1,2-oxazol-4-yl)-propanoic-acid
ANOVA	Analysis of Variance
ATP	adenosine triphosphate
CHO	Chinese Hamster Ovary
DMEM	Dulbecco’s Modified Eagle’s Medium
DMSO	Dimethyl sulfoxide
EC_50_	Efficacy
GABA	Gamma-aminobutyric acid
HEK293T	Human Embryonic Kidney 293T
hTRPM8	human Transient Receptor Potential Melastatin 8
[Ca^2+^]_IC_	Intracellular Ca^2+^
MCD	Methyl-beta-cyclodextrin
mTRPM3α2	TRPM3α2 variant of mouse Transient Receptor Potential Melastatin 3
Myr	Myriocin
PS	Pregnenolone sulphate
SEM	standard error of mean
SM	Sphingomyelins
SMase	Sphingomyelinase
TG	Trigeminal ganglion
TRP	Transient Receptor Potential
TRPA1	Transient Receptor Potential Ankryrin 1
TRPM	Transient Receptor Potential Melastatin
TRPV1	Transient Receptor Potential Vanilloid 1
